# Anesthetic Considerations in Congenital Myasthenic Syndrome: A Case Report

**DOI:** 10.7759/cureus.99454

**Published:** 2025-12-17

**Authors:** Ziyad O Knio, Joseph Dean, Joseph O'Brien

**Affiliations:** 1 Department of Anesthesiology, University of Virginia, Charlottesville, USA; 2 Department of Pediatric Anesthesiology, Virginia Commonwealth University, Richmond, USA

**Keywords:** congenital myasthenic syndrome, neuraxial analgesia, neuromuscular blockade, pediatric anesthesiology, pediatric ventilation

## Abstract

Congenital myasthenic syndrome poses unique anesthetic challenges. Most notably, this rare disease is heterogeneous. Affected individuals’ responses to depolarizing and non-depolarizing neuromuscular blocking agents are poorly understood. Additionally, while neuraxial techniques may confer respiratory benefits due to their opioid-sparing profiles, it is unclear to what extent local anesthetics may impair signal transmission at the neuromuscular junction. This case report raises concern for heightened sensitivity to non-depolarizing neuromuscular blocking agents in patients with congenital myasthenic syndrome. Neuraxial analgesia with caudal administration of dilute local anesthetics appears to be safe.

## Introduction

Congenital myasthenic syndrome is a rare disease, with a prevalence estimated at 9.2 cases per million [1]. It is characterized by genetic defects involving the neuromuscular junction. These gene mutations may result in pathophysiologic protein expression at the presynaptic, synaptic, or postsynaptic junction. Unlike autoimmune myasthenia gravis, congenital myasthenic syndrome does not involve autoantibodies to the postsynaptic acetylcholine receptor [2]. Additionally, congenital myasthenic syndrome is characteristically distinct from neonatal myasthenia gravis, as the latter is characterized by placental transfer of maternal antibodies to the acetylcholine receptor [3].

Perioperative management of patients with congenital myasthenic syndrome poses several challenges. Specifically, there is a paucity of literature documenting patient response to depolarizing and non-depolarizing neuromuscular blockade. Optimal techniques for reversal of neuromuscular blockade have not been described. Outpatient therapy varies based on the genetic subtype; therefore, not all affected patients are maintained on acetylcholinesterase inhibitors. Finally, it is unclear whether local anesthetics may affect signal transmission at the neuromuscular junction [4]. We present a case report of one infant with congenital myasthenic syndrome presenting for bilateral inguinal hernia repair and gastrostomy tube placement.

A written Health Insurance Portability and Accountability Act (HIPAA) authorization to use and disclose existing protected health information was obtained from the subject of this case report. This singleton case report did not require Institutional Review Board approval. This manuscript adheres to the applicable EQUATOR guideline (CARE guidelines for clinical case reporting) [5].

## Case presentation

A two-month-old male infant weighing 5 kg, with congenital myasthenic syndrome and arthrogryposis, presented for elective bilateral open inguinal hernia repair and concurrent gastrostomy via the open Stamm technique. 

The patient was born at 40 weeks and 2 days of gestational age by spontaneous vaginal delivery with vacuum extraction. His birth history was complicated by respiratory failure requiring intubation, with APGAR scores of 2, 6, and 7 at 1, 5, and 10 minutes of life, respectively. He ultimately required one week of mechanical ventilation, several days of continuous positive airway pressure, and one month of total intensive care. Arthrogryposis was not suspected on prenatal screening; however, his contractures and respiratory distress prompted the diagnostic panel that resulted in a RAPSN-related congenital myasthenic syndrome diagnosis. Specifically, the patient was heterozygous for two pathogenic variants in the RAPSN gene, which is consistent with a diagnosis of a biallelic autosomal recessive RAPSN-related disorder if these variants were inherited on different alleles (in trans vs in cis). Mutation testing in the biological parents has been recommended but not yet completed.

On the day of surgery, the patient’s parents reported no respiratory complications since initiation of medical therapy. The patient was started on pyridostigmine 4 to 5 mg/kg/day in four to six divided doses at one month of age. On the day of surgery, the patient was on a stable regimen of pyridostigmine, 4.8 mg per nasogastric tube, four times daily. He was last seen by pediatric neurology one month prior to surgery; his dose was adjusted for his weight at that time. This regimen was continued perioperatively. Physical exam was notable for micrognathia and contractures, with otherwise normal infant craniofacial anatomy, clear bilateral breath sounds, and no murmur auscultated on cardiac exam. This patient's American Society of Anesthesiologists physical status classification was 3. Postoperative overnight admission to the pediatric intermediate care unit was planned.

The patient was induced with 8% sevoflurane in 6 L per minute of fresh gas flow, which comprised 70% nitrous oxide and 30% oxygen. After anesthetic induction, the nitrous oxide concentration was reduced to 0%, IV access was obtained, and rocuronium 1 mg/kg was administered for intubation and surgical condition optimization. Peripheral intravenous access was challenging in the setting of arthrogryposis. The patient was intubated atraumatically by laryngoscopy with a C-MAC Miller #1 blade. A size 3.5 endotracheal tube (ETT) was secured at a depth of 11 cm at the lips. Airway management was uncomplicated despite the micrognathia. Post-intubation, a twitch monitor was applied to the ulnar nerve for train-of-four (TOF) assessment of the adductor pollicis; at this time, the TOF ratio was 0/4. Post-tetanic twitches were not measured. For analgesia, 1 mL/kg of ropivacaine 0.2% with epinephrine 1:200,000 was administered via caudal epidural single-shot injection, with no noteworthy neuromuscular interactions. The authors intended for the block to cover the hernia site but not the gastrostomy site. At 71 minutes after the induction dose of rocuronium, neuromuscular recovery was noted by the surgical team in the form of increased abdominal wall tension. An additional rocuronium 1 mg/kg was administered by the anesthesiology resident; relaxation was noted, and the surgical procedures concluded uneventfully.

**Figure 1:**
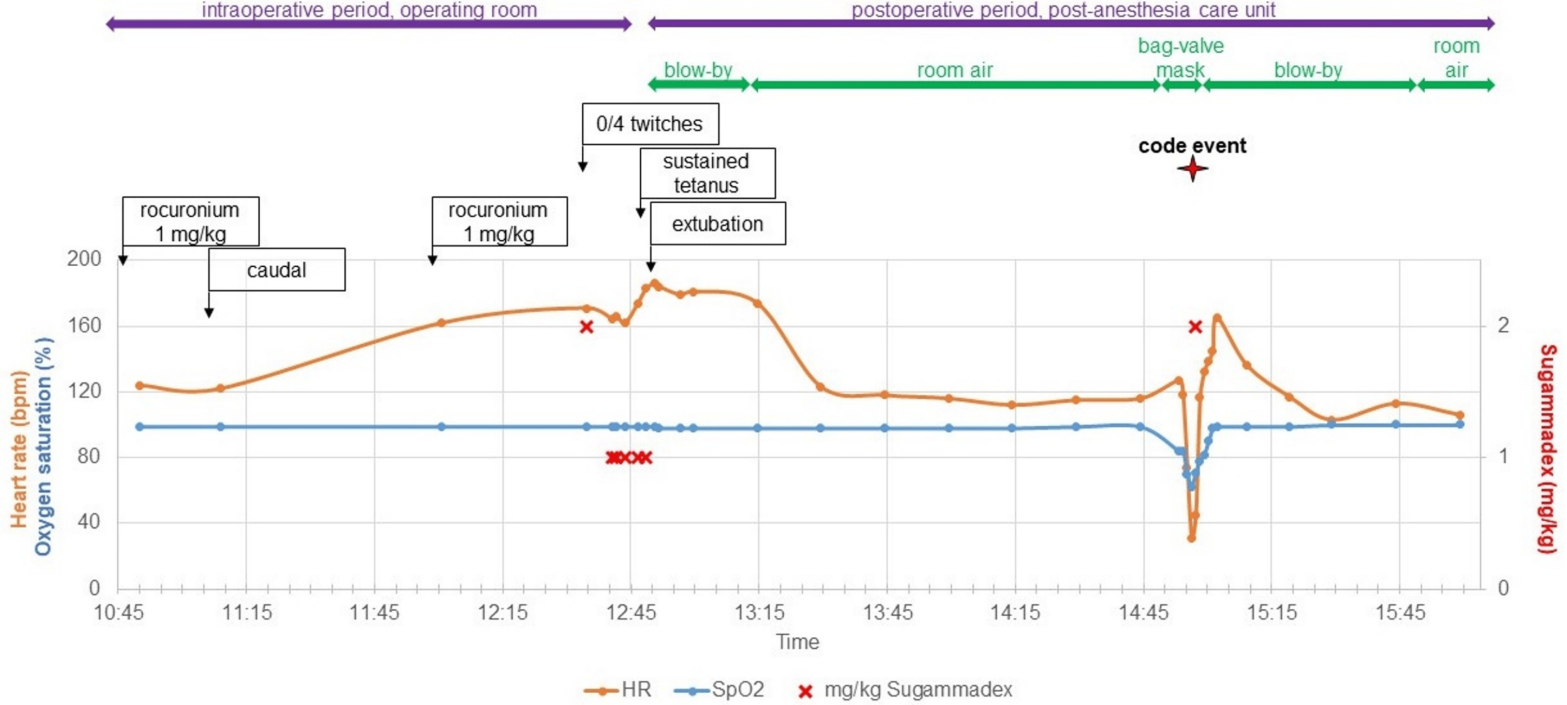
Time course of intraoperative emergence and postanesthesia care

Beginning 34 minutes after the second administration of rocuronium, attempts at neuromuscular blockade reversal began in the operating room. Initially, the patient had no response to electrocutaneous nerve stimulation (TOF 0/4). Adequate reversal required a cumulative 7 mg/kg sugammadex, titrated in 5 mg (1 mg/kg) aliquots over 13 minutes, with TOF testing performed after each aliquot. Adequate reversal was evidenced by TOF monitoring and confirmatory sustained tetanus. Extubation and emergence were otherwise unremarkable (Table 1 and Figure 1).

**Table 1:** Time course of intraoperative events and postanesthesia care PACU, post-anesthesia care unit; SpO_2_, oxygen saturation; TOF, train-of-four

Phase	Time	Events
Operating room	10:29	Inhalational induction
	10:50	Rocuronium 1 mg/kg
	10:59	TOF 0/4
	11:09	Single-shot caudal with 1mL/kg ropivacaine 0.2% and epinephrine 1:200,000
	11:23	Surgical timeout
	11:58	Hernia repair complete, begin gastrostomy
	12:01	Rocuronium 1 mg/kg
	12:35	Gastrostomy complete, TOF 0/4, sugammadex 2 mg/kg
	12:41	Residual blockade, sugammadex 1 mg/kg
	12:42	Residual blockade, sugammadex 1 mg/kg
	12:44	Residual blockade, sugammadex 1 mg/kg
	12:47	Residual blockade, sugammadex 1 mg/kg
	12:49	Residual blockade, sugammadex 1 mg/kg
	12:50	Sustained tetanus
	12:51	Extubation, PACU transport
PACU	12:57	SpO2 98% on blow-by
	13:15	SpO2 98% on room air
	14:54	SpO2 84% on room air
	14:56	Bag-valve mask ventilation initiated for hypoxia and bradycardia
	14:57	SpO2 62% and heart rate 31
	14:58	Chest compressions, sugammadex 2mg/kg
	14:59	Heart rate recovered to 117, SpO2 78% and improving
	15:02	Bag-valve mask ventilation discontinued, SpO2 98% on blow-by
	15:45	SpO2 100% on room air

While recovering in the post-anesthesia care unit, two hours post-extubation, the attending anesthesiologist was called urgently for acute hypoxia and bradycardia. The arterial oxygen saturation reached 62% (nadir), and the heart rate reached 31 beats per minute (nadir). This was treated with positive-pressure ventilation, approximately 30 seconds of chest compressions, and sugammadex 2 mg/kg, with prompt recovery of vital-sign stability and spontaneous ventilation (Table 1 and Figure 1). The patient was subsequently admitted to the pediatric intensive care unit (PICU) for monitoring. Central apnea was favored to be the most likely etiology of his respiratory failure, given the infant’s age and his acute, rather than progressive, decline. Recurarization cannot be excluded, especially in the setting of observed recovery after the administration of sugammadex.

The patient was transferred to the acute care floor on postoperative day 1. On postoperative day 2, the patient was noted to be in respiratory distress, characterized by congestion, increased work of breathing, desaturations, tachypnea, and relative bradycardia. A chest X-ray demonstrated decreased lung volumes. Oxygen was delivered via high-flow nasal cannula, and albuterol was administered, with clinical improvement. The patient was transferred back to the PICU and started on bilevel positive airway pressure (BiPAP). An infectious etiology was favored after nasopharyngeal viral testing detected rhinovirus/enterovirus. Myasthenic crisis was considered, however unlikely, given the genetic (versus autoimmune) nature of his disease. On postoperative day 3, albuterol was discontinued, and gastric tube feeds were started. BiPAP was discontinued on postoperative day 7. High-flow oxygen was discontinued on postoperative day 11. He was discharged home on room air on postoperative day 12.

## Discussion

This report describes a patient with congenital myasthenic syndrome undergoing an open bilateral inguinal hernia repair and gastrostomy tube placement, requiring neuromuscular blockade and adequate analgesia. This patient’s postoperative course was complicated by apnea, possibly secondary to recurarization after reversal with sugammadex. Concurrent viral respiratory illness further complicated his postoperative recovery.

A single case report cannot establish causal relationships or provide definitive recommendations. In the absence of more high-quality evidence and rigorously designed studies, the authors, at this time, advise against large boluses of neuromuscular blocking agents without guidance from twitch monitoring. Ready availability of sugammadex in recovery phases for these patients would be a prudent quality intervention.

Congenital myasthenic syndromes are incredibly rare; currently, the prevalence is estimated at 9.2 cases per million, though it is likely that the disease has been underdiagnosed historically [1]. This disease is heterogeneous; in fact, over 30 genes have been implicated in congenital myasthenic syndromes [1]. It is often characterized as presynaptic, synaptic, or postsynaptic [2]. Not surprisingly, phenotypes are highly variable. This patient was known to have genetic variants in the RAPSN gene, which encodes the rapsyn protein. This protein is responsible for anchoring the acetylcholine receptor at the muscle endplate. His respiratory distress at birth is fairly typical for this subtype. Prognosis and frequency of crises improve with age, and a later onset would have correlated with a more favorable prognosis [1].

The relative sensitivity to non-depolarizing neuromuscular blocking drugs in congenital myasthenic syndrome is not well-defined. While patients with autoimmune myasthenia gravis demonstrate resistance to depolarizing neuromuscular blockers and sensitivity to non-depolarizing neuromuscular blockers, this relationship cannot be extrapolated to congenital myasthenic syndrome gene defects. Perhaps sensitivity varies by genetic subtype. Of note, patients with Lambert-Eaton myasthenic syndrome demonstrate sensitivity to both depolarizing and non-depolarizing agents, suggesting that the affected neuromuscular junction site (presynaptic, synaptic, or postsynaptic) plays a significant role in individual pharmacodynamics.

Postoperative non-invasive positive-pressure ventilation is advisable for patients with congenital myasthenic syndrome, particularly those needing nightly respiratory support [6,7]. An existing case report of the perioperative management of a patient with congenital myasthenic gravis for elective cesarean section suggests that local anesthetics in high concentration may interfere with neuromuscular transmission [4]. As such, it has been recommended that these patients be administered spinal, rather than epidural, anesthesia for cases in which either is appropriate. Spinal anesthesia has been used safely in at least one other elective cesarean patient with congenital myasthenic syndrome [8]. The present case is unique in that caudal anesthesia is the preferred neuraxial technique in infants. Neuraxial analgesia was thought to confer significant benefit, as it would reduce opioid requirements and preserve respiratory function; however, the authors did acknowledge the interaction between local anesthetics and neuromuscular transmission and therefore opted to use a maximum of 1 mL/kg of dilute local anesthetic for the caudal block. Calişkan et al. (2008) describe the successful use of caudal anesthesia as an alternative to general anesthesia in a two-year-old child with congenital myasthenic syndrome undergoing orchiopexy [9].

## Conclusions

In conclusion, this case raises concern for a heightened sensitivity to aminosteroid non-depolarizing neuromuscular blocking agents in patients with congenital myasthenic syndrome. It underscores the need for cautious, monitored use of neuromuscular blockade. There is no evidence to suggest against the continuation of chronic maintenance therapy postoperatively, although the optimal pharmacological treatment does vary by genetic subtype. Neuraxial analgesia with local anesthetics appears safe after consideration of the available techniques and surgical plan.
